# Novel Megaptera novaeangliae (Humpback whale) haplotype chromosome-level reference genome

**DOI:** 10.1038/s41597-024-03922-9

**Published:** 2024-10-10

**Authors:** Maria-Vittoria Carminati, Vlonjat Lonnie Gashi, Ruiqi Li, Daniel Jacob Klee, Sara Rose Padula, Ajay Manish Patel, Andy Dick Yee Tan, Jacqueline Mattos, Nolan Kane

**Affiliations:** 1 Department of Ecology and Evolutionary Biology, University of Colorado Boulder, Boulder, CO USA; 2 The Unknown Scientist Institute, Aurora, CO USA; 3 Laboratory of Evolutionary Ecology and Plant Genomics, Department of Plant Biology, Institute of Biology, University of Campinas, São Paulo, Brazil

**Keywords:** Genome, Phylogenetics, Population genetics

## Abstract

The sequencing of a kidney sample (KW2013002) from a stranded Megaptera novaeangliae (Humpback whale) calf is the first chromosome-level reference genome for this species^1^. The calf, a 457 cm and 2,500 lbs male, was found stranded in Hawai’i Kai, HI, in 2013 and was marked as abandoned/orphaned. In 2023, 1 g of kidney was sequenced with PacBio long-read DNA sequencing, chromatin conformation capture (Hi-C), RNA sequencing, and mitochondrial sequencing to comprehensively characterize the genome and transcriptome of *M. novaeangliae*. Data validation includes a synteny analysis, mitochondrial annotation, and a comparison of BUSCO scores (scaffold v. reference genome and *Balaenoptera musculus* (Blue whale) v. *M. novaeangliae*). BUSCO analysis was performed on an *M. novaeangliae* scaffold-level assembly to determine genomic completeness of the reference genome, with a scaffold BUSCO score of 91.2% versus a score of 95.4%. Synteny analysis was performed using the *B. musculus* genome as comparison to determine chromosome-level coverage and structure. Further, a time-based phylogenetic tree was constructed using the sequenced data and publicly available genomes.

## Background & Summary

*M. novaeangliae* are significant marine organisms with a broad geographical distribution, inhabiting all major oceans from the polar regions to the tropics^2^. These whales undertake extensive migrations, traveling thousands of miles annually between feeding grounds in high-latitude waters during the summer and breeding grounds in warmer, low-latitude waters during the winter^2^. Their role in marine ecosystems is critical, as they contribute to nutrient cycling through the release of fecal plumes that stimulate the growth of phytoplankton, forming the base of the oceanic food web^3,4^. Humpback whales are known for their complex vocalizations and acrobatic behaviors, which have made them a focal point for marine tourism and research^2,3^. Their populations have experienced significant recovery following historical whaling pressures, highlighting the impact of conservation efforts. However, they continue to face threats from entanglement in fishing gear, ship strikes, and environmental changes, necessitating ongoing monitoring and protective measures.

*M. novaeangliae*, known for its cosmopolitan distribution, has historically been classified as a single species. However, recent analyses suggest the presence of several subspecies with distinct evolutionary trajectories^2^. Despite this, research into *M. novaeangliae* genetics has been limited by the absence of a complete reference genome.

Although previous efforts have yielded a scaffold-level assembly of the *M. novaeangliae* genome, this falls short of the “platinum-level” standard set by the Cetacean Genome Project (CGP) in 2020^5–7^. The scaffold-level assembly was not a chromosome-level assembly and contained gaps^6,7^. Our study aimed to bridge this gap by generating a high-quality, chromosome-level assembly of the *M. novaeangliae* genome^1,8,9^. This reference genome not only enhances our understanding of *M. novaeangliae* subspeciation but also provides valuable insights into its genetic variation, underlying cell regulation, and the evolutionary mechanisms driving its large size and cancer resistance^1^.

Using the CGP’s platinum-standard criteria, we assembled the *M. novaeangliae* genome at the chromosome level, with one sequence per chromosome in haplotype format^1,8,9^. This comprehensive assembly allowed us to conduct a range of analyses, including mtDNA annotation, a synteny analysis, generating a phylogenetic tree, comparing the genome against mammalian BUSCO databases, and gene duplication investigations. The availability of this chromosome-level reference genome will catalyze future research on *M. novaeangliae* and cetaceans, offering unprecedented opportunities to explore their genetic diversity, evolution, and conservation.

## Methods

### Sample information

A kidney sample (KW2013002) was collected from a *M. novaeangliae* calf on January 15, 2013, in Hawai’i Kai, HI, and deposited at the National Institutes of Standards and Technology (NIST). The sample was not collected by the authors so information regarding collection is limited to that presented herein. The calf, a 457 cm and 2,500 lbs male at the time of necropsy, was first observed on January 14, 2013, in shallow water and died between January 14 and January 15, 2013, via stranding. The calf was marked as abandoned/orphaned. In 2023, 1 g of KW2013002 was sampled for sequencing by Cantata Bio.

### PacBio long reads DNA sequencing

Quantification of DNA samples was performed using the Qubit 2.0 Fluorometer. For the construction of the PacBio SMRTbell library, targeting an insert size of approximately 20 kb, the SMRTbell Express Template Prep Kit 2.0 was employed following the manufacturer’s recommended protocol and default settings. The library was subsequently prepared for sequencing by binding to polymerase using the Sequel II Binding Kit 2.0 (PacBio) and loaded onto the PacBio Sequel II system. Sequencing was executed using PacBio Sequel II 8 M SMRT cells to ensure comprehensive coverage and high-quality reads.

Quality control of the extracted DNA was performed using nanodrop and gel. The OmniC library quality control was done using the Hifiasm draft assembly and showed a high amount of long-range linkage reads. The OmniC sequencing data was also quality controlled to examine Q30%, and the quality score matched the Illumina standard. The scaffolding algorithm HiRise also has a built-in quality control that uses only reads with a map score of over 40.

Chromatin was fixed *in situ* within the nucleus using formaldehyde, followed by digestion with DNase I. The processed chromatin had its ends repaired and was then ligated to a biotinylated bridge adapter, facilitating proximity ligation of adapter-containing ends. Post-proximity ligation, the crosslinks were reversed, and the DNA was purified—a critical step involved treating the purified DNA to eliminate any non-internal biotin. The sequencing libraries were prepared using NEBNext Ultra enzymes and Illumina-compatible adapters, with biotin-containing fragments isolated using streptavidin beads before PCR enrichment. Sequencing was performed on an Illumina HiSeqX platform to achieve approximately 30x coverage.

### Contig assembling and scaffolding

The *de novo* assembly process utilized PacBio CCS reads and Omni-C reads as input for HiC-Hifiasm, employing default parameters. This approach facilitated the generation of a separate de novo assembly for each haplotype, enhancing the accuracy and integrity of the genomic reconstruction.

The scaffolding phase involved the integration of the de novo assembly with Dovetail Omni-C library reads through HiRise, a software pipeline tailored for scaffolding genome assemblies using proximity ligation data. Alignment of Omni-C library sequences to the draft assembly was achieved using bwa, with the mapped read pairs analyzed by HiRise to construct a likelihood model for genomic distance. This model, along with additional information from the synteny analysis (see below), informed the identification and correction of misjoins, the scoring of potential joins, and the execution of joins exceeding a defined confidence threshold.

### Synteny analysis

The *M. novaeangliae* newly-assembled scaffolds were mapped to the *B. musculus* whole genome (GenBank GCA_009873245.3) in order to map the synteny between the two species^1,8,10^. A synteny analysis was performed using JupiterPlot 1.0^11–13^, a software tool that uses circos-based consistency plots to map a given set of scaffolds with a reference genome.

### RNA sequencing

Total RNA was extracted employing the QIAGEN RNeasy Plus Kit, adhering to the manufacturer’s instructions. Quantification of RNA involved the Qubit RNA Assay and the TapeStation 4200 system. Before library preparation, DNase treatment was applied, followed by AMPure bead cleanup and rRNA depletion using QIAGEN FastSelect -HMR. The NEBNext Ultra II RNA Library Prep Kit was used for library preparation per the manufacturer’s protocols. Sequencing of the prepared libraries was conducted on the NovaSeq 6000 platform, utilizing a 2 × 150 bp configuration to ensure comprehensive transcriptome coverage.

## Data Records

The RNA-Seq (SRX24476266), Omni-C (SRX24476265), and raw reads of the *M. novaeangliae* genome (SRX24476264) are available on the NCBI website under SRA SRP506011^9^. The mitochondrial sequence is publicly available at GenBank with accession number PP475430.1^14^. The chromosome-level genome assembly is available via GenBank at NCBI with submission number SUB14625650 and accession number JBGMDX000000000 (WGS JBGMDX010000001:JBGMDX010001355)^1^. The raw genome assembly is publicly available via Dryad^8^.

## Technical Validation

The quality of the genome assembly was evaluated using a comprehensive set of metrics and analyses. The scaffold assembly had a total length of 2.27Mbp and an N50 of 9.15 Mbp^1^,^6^,^7^. The reference input assembly had a total length of 3.15 Gbp with an N50 of 36.344 Mbp, indicating a high degree of contiguity^9^. The reference genome improved upon this, with a total length of 2.48 Gbp and a significantly higher N50 of 116.74 Mbp, suggesting a substantial increase in contiguity and completeness^1^. The N90 values also improved from 2.35 Mbp to 83.01 Mbp, indicating that a larger proportion of the genome is represented in longer scaffolds, further improving contiguity^1^. The largest scaffold for the reference genome was 191,558,633 bp, which also indicates enhanced contiguity achieved through the HiRise scaffolding process^1^. Additionally, while the input assembly had 0 gaps, the reference genome had 59 gaps, which could potentially represent regions of the genome that are difficult to assemble or highly repetitive.

Furthermore, the evaluation included an assessment of genome completeness using BUSCOs, which are conserved single-copy genes. The reference genome showed high completeness, with 98.04% of BUSCOs being complete, and 96.08% being complete and single copy. This indicates that the assembly is highly representative of the expected gene content for eukaryotic species. The use of the Dovetail Omni-C library and the HiRise scaffolding pipeline contributed to the high quality of the assembly by leveraging proximity ligation data to improve contiguity and accuracy. Overall, the genome assembly appears to be of high quality, with improved contiguity and completeness compared to the input assembly.

The resulting comparison shows that the reference genome is a chromosome-level assembly, is longer than the scaffold (2.48 Gbp v. 2.27 Gbp), has longer sequences, and is missing less data (0.0003% v. 5.4534%).

### Benchmarking Universal Single-Copy Orthologs (BUSCO) Gene Analysis Against Mammalian Genomes

BUSCO analysis was run with the mammalian ortholog dataset to compare the assembly against a set of highly conserved genes present in placental mammals. When compared to the existing scaffold, the reference genome had higher numbers of conserved BUSCO scores specific to placental mammals:

1. *M. novaeangliae* Reference Genome
  Complete BUSCOs (C): 95.4% [Single-copy: 92.7%, Duplicated: 2.7%]
  Fragmented BUSCOs (F): 1.0%
  Missing BUSCOs (M): 3.6%
  Total number of BUSCOs searched (n): 12,234
2. *M. novaeangliae Scaffold Genome*
  Complete BUSCOs (C): 91.2% [Duplicated: 0.8%]
  Fragmented BUSCOs (F): 4.8%
  Missing BUSCOs (M): 4.0%
  Total number of BUSCOs searched (n): 6,253

These readings indicate the completeness and duplication status of the genomes being analyzed, with the reference genome showing higher completeness compared to the scaffold genome. The comparison of placental mammal BUSCO scores for reference and scaffold *M. novaeangliae* genome indicates a near 50% reduction in fragmented or missing BUSCO genes (Reference: 4.6%, Scaffold: 8.8%). The percentage of duplicated BUSCOs is also provided, indicating the level of duplication in the identified complete BUSCOs.

To further validate the *M. novaeangliae* reference genome, it was compared to the *B. musculus* genome, giving the following results^1^,^8^,^10^.

1. *B. musculus*
  Complete BUSCOs (C): 95.1% [Single-copy: 92.5%, Duplicated: 2.6%]
  Fragmented BUSCOs (F): 1.4%
  Missing BUSCOs (M): 3.5%
  Total number of BUSCOs searched (n): 9,226
2. *M. novaeangliae*
  Complete BUSCOs (C): 95.4% [Single-copy: 92.7%, Duplicated: 2.7%]
  Fragmented BUSCOs (F): 1.4%
  Missing BUSCOs (M): 3.2%
  Total number of BUSCOs searched (n): 9,226

The comparison indicates that the *M. novaeanglia*e reference genome is more complete than the previous scaffold genome and is comparable to the *B. musculus* reference genome (Tables 1, 2).

**Table 1:** Summary of assembly characteristics for *M. novaeangliae* reference genomes, comparing the reference genome and the scaffold.

	Mammalian BUSCO genes			
	Single Copy Complete	Duplicated	Fragmented	Missing
*M. novaeangliae*	8,555	258	127	286
Percentage of mammalian BUSCO genes	92.70%	2.80%	1.40%	3.10%
Shared between *M. novaeangliae* and *B. musculus*	8,330	162	83	211
Percentage of *M. novaeangliae* genes shared with *B. musculus*	97.30%	62.80%	65.30%	73.80%

**Table 2:** Summary of the mammalian BUSCO analysis on the *M. novaeangliae* reference genome and a comparison between it and the closely related *B. musculus* reference genome.

*M. novaeangliae* assemblies	# of scaffolds	Total length (Gb)	Longest sequence (Mb)	N50 (Mb)	N90 (Mb)	L50	L90	Missing data (% of total length)
Reference	23	2.48	191.56	116.74	83.01	9	19	0.0003
Scaffold	2558	2.27	29.35	9.14	2.35	79	273	5.4534

A comparison within the *M. novaeangliae* and *B. musculus* BUSCO genes was then performed, with the following outcome:

### Synteny analysis

**Fig. 1:**
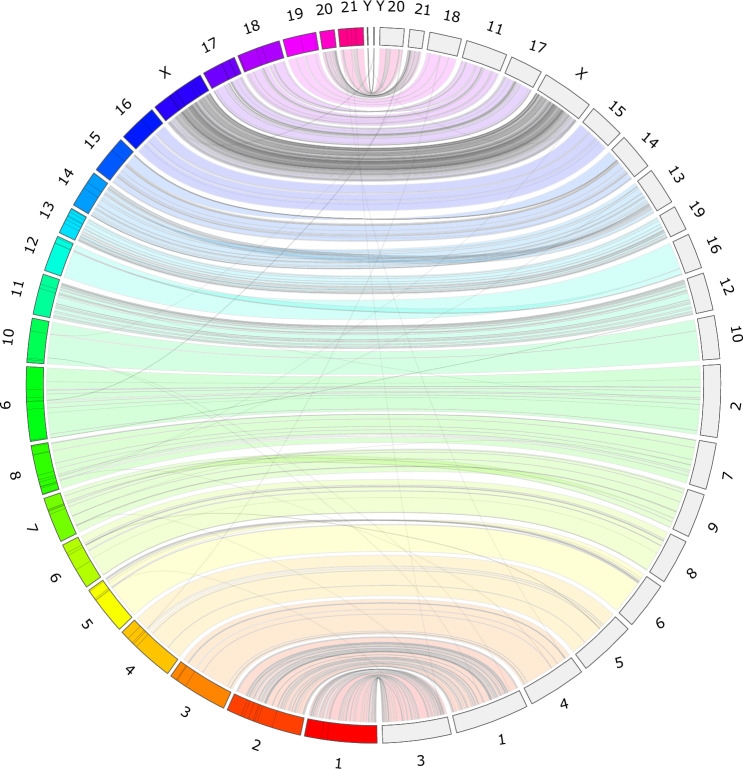
Jupiterplot of *M. novaeangliae* and *B. musculus* genomes. This figure illustrates the overall alignment of these two genomes, identifying syntenic regions between their chromosomes. Connected regions show the genetic links between both genomes.

Synteny analysis involves comparing the arrangement of genes or genetic elements in different genomes to understand their evolutionary relationships and identify conserved regions. By using Jupiter Plot we identified and visualized these syntenic regions between the humpback whale genome and the blue whale genome The synteny plot (Fig. 1) shows the syntenic regions between these two genomes, and a high degree of collinearity between them. The X and Y chromosomes are also syntenic, showing a high level of consistency in the evolutionary relationships between these two species.

### Mitochondrial DNA analysis

**Fig. 2:**
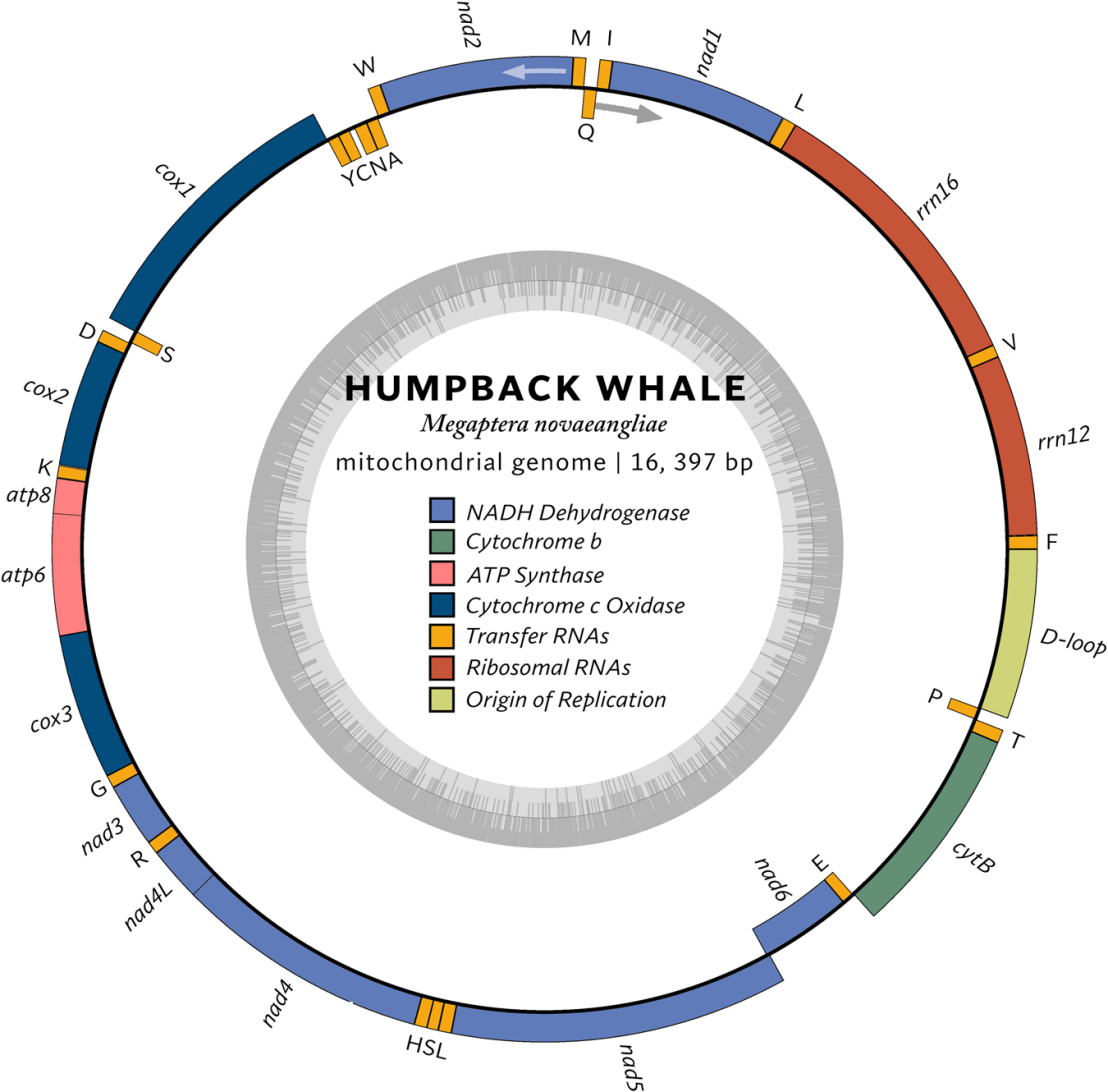
Annotation of the circular mitochondrial genome.

Our study also analyzed the mitochondrial genome of *M. novaeangliae*^14^. The complete *M. novaeangliae* genome was sequenced and assembled (Fig. 2). The genome contains the typical set of mitochondrial genes found in vertebrates, including genes coding for ribosomal RNAs, transfer RNAs, and protein-coding genes such as NADH dehydrogenase subunits, cytochrome c oxidase subunits, ATP synthase subunits, and cytochrome b.12,13 The genome also contains a D-loop region of 915 bp, which is a non-coding region often used for studying genetic diversity and population structure. Annotation was done by chloroblox/mitos and visualization was done on chlorobox. This sequence represents an important resource for future studies on the evolution, population genetics, and conservation of humpback whales.

### Phylogenomic analysis

**Fig. 3:**
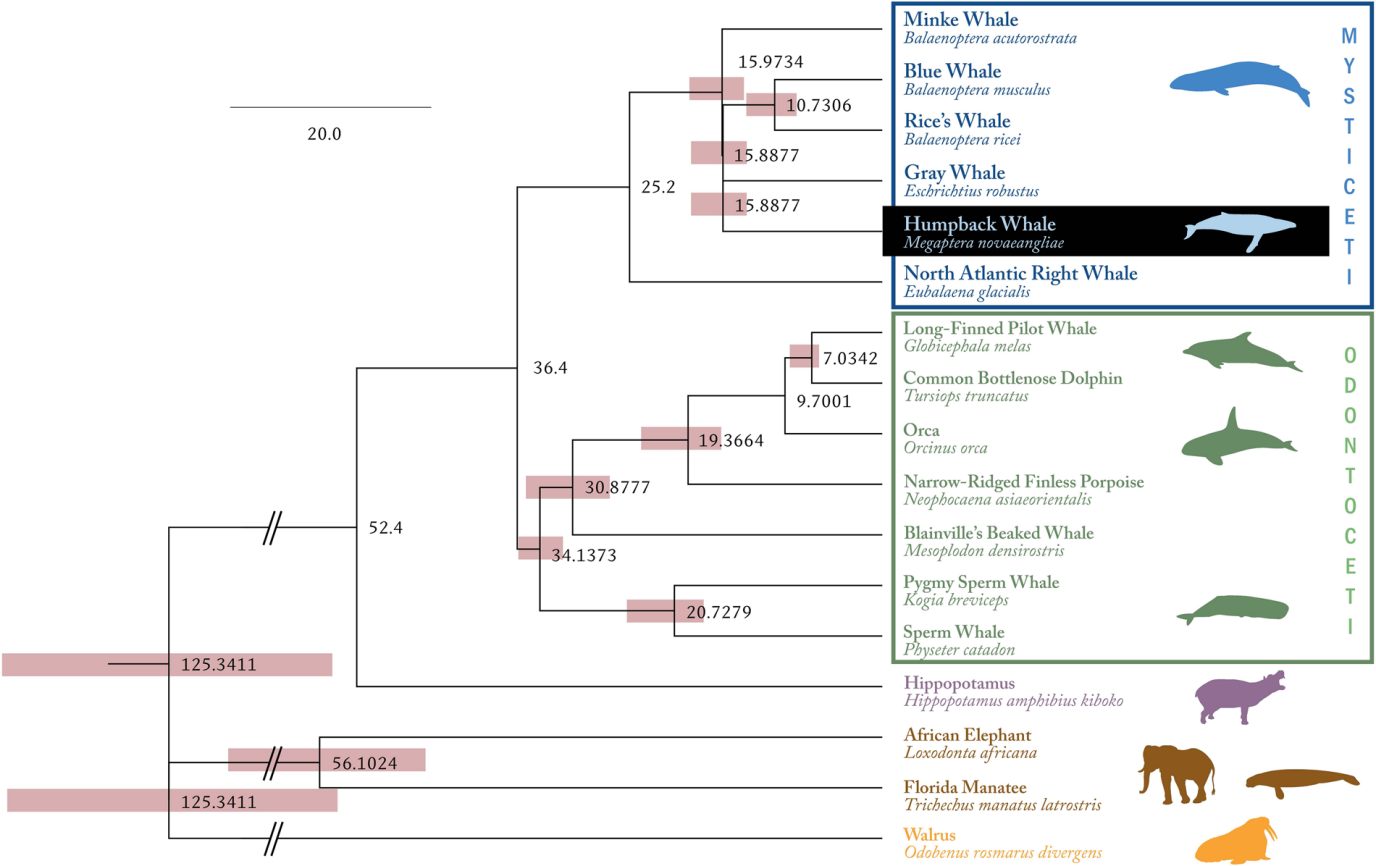
The phylogenomic tree’s topology was congruent with the recent phylogeny from target capture data. It identified *E. robustus* (gray whale) as the closest relative to *M. novaeangliae*.

Protein coding genes were retrieved from 16 mammal genomes downloaded from NCBI (Fig. 3)^10,15–29^. Along with genes in the *M. novaeangliae* genome, they were clustered into gene families using OrthoFinder v2.27^30^. The sequences from single copy gene families were aligned with mafft v7.508^10^, then trimmed with TrimAL v1.4 (http://trimal.cgenomics.org/). The trimmed alignments were concatenated to a super-matrix^11,31^. IQtree2 was used to infer phylogenetic trees from the super-matrix with 1000 bootstraps^32^. ModelFinder integrated in the IQtree2 was employed to select the best-fit model. Then the phylogeny was time calibrated using LSD2 embedded in IQtree2 with fossil records and inferred divergent dates (Fig. 3)^33^.

The protein-coding proteins were annotated with eggnog mapper against Gene Ontology (GO), KEGG, EC, CAZy, databases with default settings. 28,109 gene families were identified using OrthoFinder, of which 511 were single copies^34^. The final concatenated super-matrix was 240,833 bp. The best-fit model is Q.bird + F + I + R6 according to BIC. Among the total of 38660 genes, 21159 (54.73%) were annotated in at least one of the emapper databases. Specifically, 17,574 (45.46%) have gene names, 13,871 (35.88%) feature GO annotations, 3,196 (8.27%) are associated with EC numbers, and 13,461 (34.82%) are linked to KEGG pathways.

## Data Availability

The data analyses were performed according to the manuals and protocols provided by the developers of the respective bioinformatics tools and no custom code was used in the execution of the study. All software and codes used in this work are publicly accessible, and their corresponding versions are specified in the Methods section.
